# Treatment Invasiveness and Illness Perceptions Are Strongly Associated With Outcome Expectations in Patients Treated for Hand or Wrist Conditions: A Cross-sectional Study

**DOI:** 10.1097/CORR.0000000000002540

**Published:** 2023-01-24

**Authors:** Willemijn Anna de Ridder, Lisa Hoogendam, Fadoua Zeroual, Harm Pieter Slijper, Robbert Maarten Wouters, Guus Maarten Vermeulen, Ruud Willem Selles, Mark Johannes Willem van der Oest

**Affiliations:** 1 Department of Plastic, Reconstructive, and Hand Surgery, Erasmus MC, Rotterdam, the Netherlands; 2 Department of Rehabilitation Medicine, Erasmus MC, Rotterdam, the Netherlands; 3 Hand and Wrist Center, Xpert Clinics, Eindhoven, the Netherlands; 4 Center for Hand Therapy, Xpert Handtherapie, Eindhoven, the Netherlands; 5 Department of Intensive Care, Erasmus Medical Centre, Rotterdam, the Netherlands

## Abstract

**Background:**

Multiple studies have shown that more-positive outcome expectations are associated with better treatment outcomes. Although this has not been shown to represent a causal relationship, there nonetheless is an interest in positively modifying outcome expectations to improve treatment outcomes. However, little is known about what is independently associated with outcome expectations in clinical practice. For example, it is unknown to what extent expectations are associated with treatment or patient characteristics such as sociodemographics or with patient-reported outcome measures (PROMs) on patient perceptions of physical or mental health or illness. Studying factors associated with outcome expectations may provide relevant information for clinicians and researchers aiming to improve outcome expectations. Improving expectations might, in turn, improve treatment outcomes.

**Question/purpose:**

Which factors (that is, sociodemographics, PROMs, illness perceptions, treatment, surgeon, and location) are independently associated with outcome expectations in patients with hand or wrist conditions?

**Methods:**

This was a cross-sectional study. Between July 2018 and December 2021, we screened 21,327 patients with a diagnosed hand or wrist condition with complete baseline sociodemographic data such as age and workload. Sixty percent (12,765 of 21,327) of patients completed all relevant PROMs. We excluded patients receiving rare treatments, leaving 58% (12,345 of 21,327) for inclusion in the final sample. Those who participated were more often scheduled for surgical treatment and had higher expectations. We performed a multilevel analysis involving two steps. First, we evaluated whether patients receiving the same treatment, being counseled by the same surgeon, or being treated at the same location have more similar outcome expectations. We found that only patients receiving the same treatment had more similar outcome expectations. Therefore, we used a multilevel regression model to account for this correlation within treatments, and added treatment characteristics (such as nonsurgical versus minor or major surgery) to potential explanatory factors. Second, in the multilevel hierarchical regression analysis, we added sociodemographics (Model 1), PROMs for physical and mental health (Model 2), illness perceptions (Model 3), and treatment characteristics (most-definitive model) to assess the explained variance in outcome expectations per step and the relative association with outcome expectations.

**Results:**

Sociodemographic factors such as age and workload explained 1% of the variance in outcome expectations. An additional 2% was explained by baseline PROMs for physical and mental health, 9% by illness perceptions, and 18% by treatment characteristics, resulting in an explained variance of 29% of the most-definitive model. A large number of patient and treatment characteristics were associated with outcome expectations. We used standardized betas to compare the magnitude of the effect of the different continuous and categorical variables. Among the associated variables, minor surgery (standardized beta [β] = 0.56 [95% confidence interval 0.44 to 0.68]; p < 0.001) and major surgery (β = 0.61 [95% CI 0.49 to 0.73]; p < 0.001) had the strongest positive association with outcome expectations (receiving surgery is associated with higher outcome expectations than nonsurgical treatment). A longer illness duration expected by the patient (-0.23 [95% CI -0.24 to -0.21]; p < 0.001) and being treated for the same condition as before (-0.08 [95% CI -0.14 to -0.03]; p = 0.003) had the strongest negative association with outcome expectations.

**Conclusion:**

Outcome expectations are mainly associated with the invasiveness of the treatment and by patients’ illness perceptions; patients before surgical treatment have more positive expectations of the treatment outcome than patients before nonsurgical treatment, even after accounting for differences in clinical and psychosocial profiles. In addition, patients with a more-positive perception of their illness had more-positive expectations of their treatment. Our findings suggest expectation management should be tailored to the specific treatment (such as surgical versus nonsurgical) and the specific patient (including their perception of their illness). It may be more beneficial to test and implement expectation management strategies for nonsurgical treatments such as physical therapy than for surgical treatments, given that our findings indicate a greater need to do so. An additional advantage of such a strategy is that successful interventions may prevent converting to surgical interventions, which is a goal of the stepped-care principles of standard care. Future studies might investigate the causality of the association between pretreatment expectations and outcomes by performing an experimental study such as a randomized controlled trial, in which boosting expectations is compared with usual care in nonsurgical and surgical groups.

**Level of Evidence:**

Level III, prognostic study.

## Introduction

Patients have expectations at the beginning of their treatments regarding potential outcomes. Several studies have shown these expectations play an important role in treatment outcomes [1, 5, 6, 27]. Although some studies suggested expectations of medical treatments are already too high and should be tempered by the clinician to cultivate realistic expectations for the patient [13, 19, 24, 35], several meta-analyses have found that patients with more-positive pretreatment expectations achieve better outcomes [1, 5, 6, 27]. Additionally, in patients treated for hand or wrist conditions, more-positive expectations have been reported to be associated with better outcomes [8, 23, 33]. In addition, positive expectations of the treatment outcomes are considered a key mechanism of placebo effects [20, 30]. The placebo effect, or contextual nonspecific effect, is a psychobiological effect that is attributed to the overall therapeutic context [25, 28]. This context can consist of patient-specific and clinician-specific factors, and the interaction of patient, clinician, treatment location, and treatment factors [12]. Clinical trials have shown considerable improvement in patients in placebo groups compared with an active or no treatment group [3, 37]. Although positive expectations increase the contextual, nonspecific effects of a treatment, expectations may vary across patients and may depend on the type of treatment the patient is about to undergo. For example, previous studies showed that patients with hand or wrist disorders scheduled for surgery have higher expectations than similar patients scheduled for nonsurgical treatment [17, 36].

### Rationale

Using the contextual effects of a treatment may improve healthcare. Because the contextual nonspecific effect is believed to work through positive expectations of the outcome of a treatment, boosting expectations might be an important part of delivering high-quality care. However, little is known about factors independently associated with patient outcome expectations in clinical practice. Knowing the independent factors associated with outcome expectations may help clinicians to improve expectations. Improving expectations might, in turn, improve treatment outcomes. Moreover, it may inform future studies in the development of interventions that boost expectations.

Therefore, we asked: Which factors (such as sociodemographics, patient-reported outcome measures [PROMs], illness perceptions, treatment, surgeon, and location) are independently associated with outcome expectations in patients with hand or wrist conditions?

## Patients and Methods

### Study Design

This was a cross-sectional study using a population-based sample of patients with hand or wrist conditions treated at our institution, and was reported following the STrengthening the Reporting of Observational studies in Epidemiology statement [34].

### Setting

Data collection was part of usual care and occurred between July 2018 and December 2021 at Xpert Clinics. Xpert Clinics currently comprises 25 clinics for hand surgery and hand therapy in the Netherlands. Twenty-three surgeons are certified by the Federation of European Societies for Surgery of the Hand, and more than 150 hand therapists are employed at our treatment centers. Xpert Clinics offers insured care for hand and wrist conditions with no access restrictions because it is covered by public health insurance. At Xpert Clinics, outcomes are routinely evaluated [29]. After a diagnosis is registered during the first consultation, a measurement track is activated, and PROM forms are emailed to the patient. All data are digitally collected using GemsTracker electronic data capture tools (GemsTracker 2020, Erasmus MC and Equipe Zorgbedrijven), a secure internet-based application for distributing questionnaires and forms during clinical research and quality registrations. More details of the procedure at Xpert Clinics have been published [29].

### Participants

Participants were eligible for inclusion if they were adults treated for a hand or wrist condition during the study period. We included patients from all measurement tracks but excluded rare treatments with fewer than 20 patients for generalizability. Treatments can be divided into nonsurgical treatments (such as orthotics, exercise therapy, or injections), minor surgery (including trigger finger release or de Quervain release), and major surgery (such as trapeziectomy with or without ligament reconstruction and tendon interposition for osteoarthritis of the thumb base or corrective osteotomy for radius malunions). Additionally, we excluded patients who did not complete all relevant questionnaires. The number of patients treated during the study period determined the sample size.

**Fig. 1:**
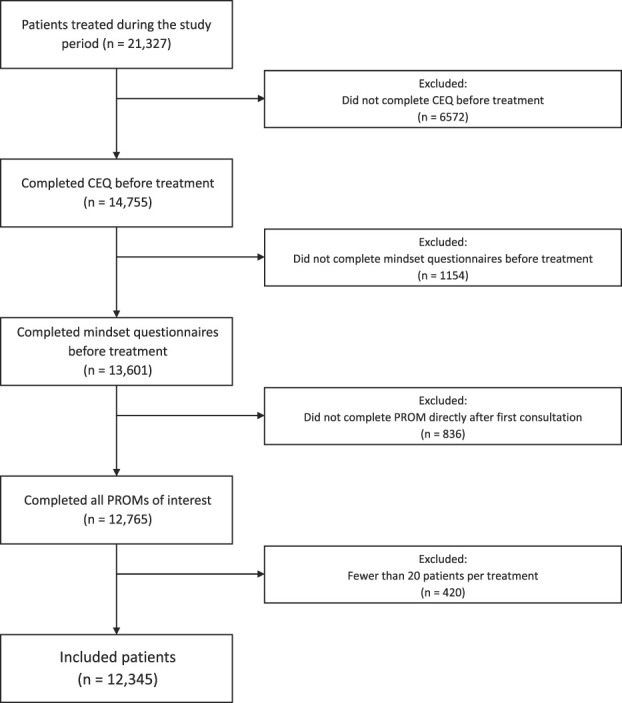
This flowchart represents the patients who were included in this study. CEQ = Credibility and Expectancy Questionnaire.

We screened 21,327 patients with complete baseline sociodemographic data such as age and workload. Sixty percent (12,765 of 21,327) of patients completed all relevant PROMs. Finally, we excluded patients receiving rare treatments, leaving 58% (12,345 of 21,327) for inclusion in the final sample (Fig. 1). To assess potential selection bias, we performed two nonresponder analyses. For this, we used the standardized mean difference as a measure of imbalance (standardized mean difference > 0.2 is considered to be imbalanced [4]). First, we compared the sociodemographic characteristics of patients who completed the Credibility and Expectancy Questionnaire (CEQ) (defined as responders) with patients who did not (defined as nonresponders). Second, we compared sociodemographic characteristics and the CEQ expectancy score of patients who additionally completed the other questionnaires of interest (responders) with patients who did not (nonresponders). In the first analysis, we found a small difference between responders and nonresponders in treatment group (standardized mean difference = 0.43) (Supplemental Table 1; http://links.lww.com/CORR/B12). In the second analysis, we found a small difference in treatment group (standardized mean difference = 0.28) and CEQ expectancy score (standardized mean difference = 0.21) (Supplemental Table 2; http://links.lww.com/CORR/B13). Those who participated were more likely to be in the surgical treatment group and to have higher expectations.

To assess the association between different degrees of surgical invasiveness, we distinguished nonsurgical treatment (such as hand therapy for thumb-base osteoarthritis), minor surgery (such as trigger finger release), and major surgery (such as triangular fibrocartilage complex reinsertion). Twenty-nine percent (3544 of 12,345) of the final sample were scheduled for nonsurgical treatment, 49% (6022 of 12,345) for a minor surgical intervention, and 23% (2779 of 12,345) for a major surgical intervention (Table 1). The number of surgical patients in the present study does not reflect the actual distribution of surgical versus nonsurgical patients at Xpert Clinics, because the inclusion of patients in the present study depends on whether a measurement track is assigned. At the time of this study, no measurement tracks were started in our cohort in patients with, for example, a “wait and see” policy or patients receiving steroid injections. Therefore, the proportion of surgical patients is overestimated in this study. Patients in the major surgery group had a longer duration of symptoms and were more often treated for the same disease previously. Patients in the minor surgery group had the most positive expectations (Supplemental Table 3; http://links.lww.com/CORR/B14). Furthermore, to assess potential differences between patients scheduled for nonsurgical treatment and patients scheduled for surgical treatment, we stratified patients into two treatment groups: nonsurgical and surgical. Seventy-one percent (8801 of 12,345) were scheduled for either minor or major surgery.

**Table 1.** Characteristics of the included patients (n = 12,345)

Characteristic	Total
Age in years, mean ± SD	55 ± 15
Female sex, % (n)	65 (7986)
Duration of symptoms in months, median (IQR)	8 (4 to 18)
Hand dominance, % (n)	
Right	89 (10,960)
Left	8 (1013)
Both	3 (372)
Occupational intensity, % (n)	
Not employed	37 (4553)
Light (working in an office)	28 (3506)
Moderate (working in a shop)	25 (3110)
Severe (working in construction)	10 (1176)
Second opinion, % (n)	2 (301)
Recurrent disease, % (n)	8 (1028)
Treatment group, % (n)	
Nonsurgical treatment	29 (3544)
Minor surgery	49 (6022)
Major surgery	23 (2779)

Nonsurgical treatments include orthotics, exercise therapy, injections; minor surgery includes minor surgical interventions such as trigger finger release or de Quervain release; major surgery includes more invasive interventions such as trapeziectomy with or without ligament reconstruction and tendon interposition for thumb-base osteoarthritis or corrective osteotomy for radius malunions.

### Variables and Measurements

The primary outcome in this study was patients’ outcome expectations of the treatment. We measured outcome expectations with the expectancy subscale of the CEQ [10]. This subscale consists of three items measuring the expected magnitude of improvement because of the prescribed treatment. Summed scores range from 3 to 27, where a higher score reflects a more positive treatment outcome expectation.

### Independent Variables

We believed patients receiving the same treatment, counseled by the same surgeon, or treated at the same location might have more similar outcome expectations than other patients. To evaluate this, we used multilevel regression modeling with a random intercept and no fixed factors and intraclass correlation coefficients (ICC). Only for treatment, we found that patients were more similar in outcome expectations (Supplemental Digital Content 1; http://links.lww.com/CORR/B15). Therefore, we included the treatment level in all subsequent analyses.

### Patient Characteristics

We divided patient characteristics into three subcategories: sociodemographics, PROMs for physical and mental health, and illness perception. Sociodemographic characteristics included age, sex (not gender, because we collect sex at the Dutch Citizen Service Administration, and we did not want to make unsupported assumptions), therapist-reported duration of symptoms (in months), hand dominance, therapist-reported occupational intensity (unemployed or light, moderate, or heavy physical labor), whether the patient visited the clinic for a second opinion, and whether the disease was recurrent (measured by the question: “Have you been treated for the same disease before?”; the answer yes would be coded as recurrent. This means that a patient answering “yes” had the same or a different treatment for the same disease previously.).

PROMs for physical and mental health included pain, hand function, health-related quality of life, psychological distress, and pain catastrophizing at baseline. We used a VAS score (range 0 to 100) to measure the mean pain as experienced in the preceding week (higher scores indicate more pain) and hand function (higher scores indicate better function). The VAS is a validated and widely used tool for measuring these constructs [14]. We measured health-related quality of life using the VAS of the EuroQol-5 Dimensions (EQ-5D) self-rated health questionnaire as an indication of the overall perceived health status (range 0 to 100; higher scores indicate better perceived health) [15, 18]. Psychological distress was measured with the Patient Health Questionnaire-4 (range 0 to 12; higher scores indicate more distress [21]), and pain catastrophizing was measured with the Pain Catastrophizing Scale (range 0 to 52; higher scores indicate a higher amount of catastrophizing [31]).

The last set of patient characteristics concerned illness perception as measured with the Brief Illness Perception Questionnaire [2, 7]. The Brief Illness Perception Questionnaire measures patients’ perception of their illness across eight domains (consequences, timeline, personal control, treatment control, identity, concern, coherence, and emotional response). Each domain is assessed with a single question (range 0 to 10; higher scores indicate more negative illness perceptions except for personal control, treatment control, and coherence, where the reverse is true) [2]. We excluded the domain of treatment control (“How much do you think your treatment can help your illness?”) because of conceptual overlap with outcome expectations.

### Treatment Characteristics

The treatment characteristics concerned the invasiveness and past effectiveness of the treatment. As an indicator of invasiveness, we coded a treatment as nonsurgical, minor surgery, or major surgery. In addition, as a proxy for the influence of the clinician’s explanation of treatment effectiveness, for each treatment, we calculated the mean improvement in function achieved in patients treated previously, using VAS function scores (-100 = maximum deterioration in function; 100 = maximum improvement in function) administered at baseline and at 3 months. We did the same for pain (-100 = maximum deterioration in pain; 100 = maximum improvement in pain).

Finally, we used the Patient-Reported Experience Measure to measure the patient’s experience with healthcare delivery, directly after the first consultation. This questionnaire is based on the Consumer Quality Index [9]. The Patient-Reported Experience Measure comprises 16 questions rated on a 4-point Likert scale, including questions about accessibility, reception in the clinic, and communication of the physician.

### Ethical Approval

Ethical approval for this study was obtained from the medical ethics committee of the Erasmus MC Medical Centre, Rotterdam (MEC-2018-1088). Informed consent was obtained from patients before data collection started.

### Statistical Methods

We used multilevel hierarchical regression analyses to test the relative association of specific patient and treatment characteristics with outcome expectations. In a hierarchical regression analysis, a set of variables is entered into a specific sequence to illustrate each set’s added amount of explained variance. This means that variables that add no or little to the explained variance remain in the model. In the first model, we entered all sociodemographic patient characteristics (such as sex, age, and occupational intensity). We added PROMs for physical and mental health (such as quality of life, pain, function, and psychological distress) in the second model, illness perceptions in the third model, and treatment characteristics in the most-definitive model (the fourth model). An advantage of hierarchical regression is that because of shared variance, some variables might be pushed out of significance when entering the next step. Consequently, only variables that are truly associated with outcome expectations remain significant in the final model. For each model, the explained variance using multilevel partitioning was calculated. Finally, we performed a stratified analysis to compare differences between factors associated with outcome expectations between patients scheduled for nonsurgical treatment and those scheduled for surgical treatment. Stratification is a useful strategy to identify interactions between subgroups such as treatment type.

A variance inflation factor greater than 3 was considered to indicate multicollinearity [22]. Based on the variance inflation factors (the highest-variance inflation factor in the multilevel hierarchical regression model equaled 2.05; in the stratified nonsurgical model, it equaled 2.12; and in the stratified surgical model, it equaled 2.03), we did not find any indication for multicollinearity in our models.

For all analyses, a p value < 0.05 was considered statistically significant. We used R statistical software version 4.1.1 for the analyses.

## Results

### Factors Independently Associated With Outcome Expectations

In our most-definitive model, we found an explained variance of 29% (Table 2). When analyzing the separate steps of the different models, sociodemographics alone provided an explained variance of 1% in outcome expectations. PROMs for physical and mental health added 2% to the explained variance. Illness perceptions (9%) and treatment characteristics (18%) explained the largest amount of variance in outcome expectations.

**Table 2.** Random effects and explained variance expressed in the marginal r2 for the most-definitive model with all treatments, nonsurgical treatments, and surgical treatments.

Random effects	Expectations for all treatments	Expectations for nonsurgical treatment	Expectations for surgical treatment
σ^2^	0.67	0.75	0.85
τ_00treatment_	0.02	0.00	0.02
ICC	0.03	0.01	0.02
N_treatment_	56	17	39
Observations	12,345	3544	8801
Marginal or conditional r^2^	0.293/0.314	0.252/0.256	0.137/0.154

**Fig. 2:**
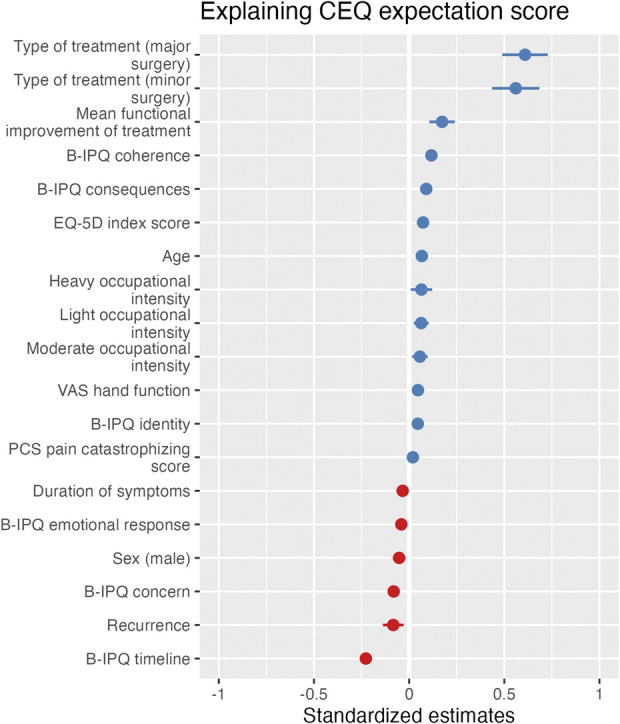
Standardized regression coefficients of the hierarchical multilevel regression model explain outcome expectations. Only significant variables are shown. EQ-5D = EuroQol-5 Dimensions; B-IPQ = Brief Illness Perception Questionnaire; PCS = Pain Catastrophizing Scale; PHQ = Patient Health Questionnaire. A color image accompanies the online version of this article.

We used standardized betas to compare the magnitude of the effect of the different continuous and categorical variables. Higher outcome expectations were associated with the following sociodemographic variables (Fig. 2) (arranged from the largest to the smallest standardized beta coefficients): higher age (0.07; p < 0.001), occupational intensity (heavy: 0.06; p = 0.02, light: 0.06; p = 0.002, moderate: 0.06; p = 0.008), shorter duration of symptoms (0.03; p < 0.001); female sex (0.05; p = 0.002), and not having been treated for the same condition before (0.08; p = 0.003) (Table 3). Higher outcome expectations were associated with the following baseline PROMs for physical and mental health (largest to smallest standardized beta coefficients): a higher EQ-5D self-rated health score (0.07; p < 0.001), better hand function (0.05; p < 0.001), and more pain catastrophizing (0.02; p = 0.048). Six of seven illness perception items were associated with greater outcome expectations (from largest to smallest): shorter illness duration expected by the patient (-0.23; p < 0.001), better understanding of the condition by the patient (0.12; p < 0.001), the more the illness affects the patient’s life (0.09; p < 0.001), less concern about the illness the patient experiences (-0.08; p < 0.001), a larger number of symptoms the patient views as being part of their illness (0.05; p < 0.001), and the less the illness affects the patient emotionally (-0.04; p < 0.001). The largest standardized beta coefficients were for treatment characteristics: major surgical treatment (0.61; p < 0.001) and minor surgical treatment (0.56; p < 0.001). This means that being at the start of a major surgical treatment increases the outcome expectations by 2.75 points (95% confidence interval 2.21 to 3.29; p < 0.001) compared with being at the start of a nonsurgical treatment (Supplemental Table 4; http://links.lww.com/CORR/B16). The mean functional improvement of the treatment was also associated with outcome expectations (0.17; p < 0.001) (Table 3).

**Table 3.** Most-definitive model (standardized beta coefficients) after the hierarchical linear regression analyses (n = 12,345) using sociodemographics, PROMs for physical and mental health, illness perception, and treatment characteristics explaining outcome expectations

	Expectations for all treatments		Expectations for nonsurgical treatment		Expectations for surgical treatment	
Variables	Standardized coefficients (95% CI)	p value	Standardized coefficients (95% CI)	p value	Standardized coefficients (95% CI)	p value
*Sociodemographics*						
Age in years	0.07 (0.05 to 0.09)	< 0.001	0.07 (0.04 to 0.11)	< 0.001	0.08 (0.06 to 0.11)	< 0.001
Sex (female)	0.05 (0.02 to 0.09)	0.002	0.09 (0.03 to 0.16)	0.01	0.05 (0.0 to 0.09)	0.04
Light occupational intensity (reference: not employed)	0.06 (0.02 to 0.10)	0.002	0.08 (0.00 to 0.16)	0.04	0.06 (0.00 to 0.11)	0.04
Moderate occupational intensity (reference not employed)	0.06 (0.02 to 0.10)	0.01	0.09 (0.01 to 0.17)	0.03	0.04 (-0.02 to 0.09)	0.18
Heavy occupational intensity (reference not employed)	0.06 (0.01 to 0.12)	0.02	0.05 (-0.07 to 0.16)	0.43	0.09 (0.01 to 0.16)	0.02
Second opinion: No	0.06 (-0.04 to 0.16)	0.21	0.06 (-0.14 to 0.26)	0.57	0.04 (-0.08 to 0.17)	0.49
Duration of symptoms in months	-0.03 (-0.05 to -0.02)	< 0.001	-0.04 (-0.07 to -0.01)	0.004	-0.03 (-0.05 to -0.01)	0.01
Right hand dominance (reference: left)	-0.01 (-0.06 to 0.04)	0.7	-0.04 (-0.15 to 0.07)	0.49	-0.01 (-0.08 to 0.06)	0.78
Both hand dominance (reference: left)	-0.03 (-0.13 to 0.07)	0.56	-0.07 (-0.26 to 0.13)	0.5	-0.02 (-0.15 to 0.11)	0.77
Recurrent: yes	-0.08 (-0.14 to -0.03)	0.003	-0.12 (-0.28 to 0.05)	0.17	-0.11 (-0.18 to -0.05)	0.001
*PROMs for physical and mental health*						
VAS function	0.05 (0.03 to 0.06)	< 0.001	0.03 (0.00 to 0.06)	0.05	0.06 (0.04 to 0.09)	< 0.001
VAS pain	0.01 (-0.01 to 0.03)	0.21	0.01 (-0.02 to 0.05)	0.41	0.01 (-0.01 to 0.04)	0.29
EQ-5D self-rated health	0.07 (0.05 to 0.09)	< 0.001	0.09 (0.05 to 0.13)	< 0.001	0.07 (0.04 to 0.10)	< 0.001
Pain Catastrophizing Score	0.02 (0.00 to 0.04)	0.048	0.01 (-0.02 to 0.05)	0.46	0.03 (0.00 to 0.05)	0.03
PHQ Depression Score	-0.01 (-0.04 to 0.01)	0.18	-0.04 (-0.09 to -0.00)	0.04	0.00 (-0.02 to 0.03)	0.74
PHQ Anxiety Score	0.01 (-0.01 to 0.03)	0.3	0.08 (0.04 to 0.12)	< 0.001	-0.03 (-0.05 to 0.00)	0.06
*Illness perception*						
B-IPQ Consequences	0.09 (0.07 to 0.11)	< 0.001	0.11 (0.07 to 0.15)	< 0.001	0.10 (0.07 to 0.13)	< 0.001
B-IPQ Timeline	-0.23 (-0.24 to -0.21)	< 0.001	-0.36 (-0.39 to -0.32)	< 0.001	-0.21 (-0.24 to -0.19)	< 0.001
B-IPQ Personal control	0.01 (-0.01 to 0.02)	0.45	0.13 (0.10 to 0.16)	< 0.001	-0.05 (-0.07 to -0.03)	< 0.001
B-IPQ Identity	0.05 (0.03 to 0.06)	< 0.001	0.03 (-0.01 to 0.06)	0.15	0.06 (0.04 to 0.09)	<0.001
B-IPQ Concern	-0.08 (-0.10 to 0.06)	< 0.001	-0.06 (-0.10 to -0.02)	0.002	-0.10 (-0.13 to -0.07)	< 0.001
B-IPQ Coherence	0.12 (0.10 to 0.13)	< 0.001	0.12 (0.09 to 0.15)	< 0.001	0.13 (0.11 to 0.15)	< 0.001
B-IPQ Emotional response	-0.04 (-0.06 to -0.02)	< 0.001	-0.02 (-0.06 to 0.02)	0.23	-0.07 (-0.10 to -0.04)	< 0.001
*Treatment characteristics*						
Type treatment (minor surgery)	0.56 (0.44 to 0.68)	< 0.001	NA	NA	NA	NA
Type treatment (major surgery)	0.61 (0.49 to 0.73)	< 0.001	NA	NA	0.01 (-0.11 to 0.12)	0.92
Mean improvement pain	-0.05 (-0.11 to 0.02)	0.167	-0.02 (-0.08 to 0.05)	0.62	-0.02 (-0.09 to 0.05)	0.59
Mean improvement function	0.17 (0.11 to 0.24)	< 0.001	0.15 (0.09 to 0.22)	< 0.001	0.11 (0.04 to 0.18)	0.002

Standardized beta coefficients, 95% CIs, and p values are displayed for the most-definitive model with all treatments, nonsurgical treatments, and surgical treatments. Standardized β coefficients, converted to the same scale, are reported to allow easier between-variable comparisons and to determine the relative association of each explanatory variable. EQ-5D = EuroQol-5 Dimensions; PROM = patient-reported outcome measures; B-IPQ = Brief Illness Perception Questionnaire; PCS = Pain Catastrophizing Scale; PHQ = Patient Health Questionnaire.

Analyzing differences in variables between the different steps of the model, we found only one difference (Supplemental Table 5; http://links.lww.com/CORR/B17). In Model 1, visiting the clinic for a second opinion was associated with lower expectations, but after adding PROMs for physical and mental health, there was no association. This implies that one (or more) of the PROMs, such as pain catastrophizing, have a shared variance with a second opinion and pushes the variable second opinion out of significance.

### Differences Between Patients Scheduled for Nonsurgical Treatment and Those Scheduled for Surgical Treatment

In the most-definitive model, including sociodemographics, PROMs for physical and mental health, illness perception, and treatment characteristics, we found an explained variance of 25% for outcome expectations of patients scheduled for nonsurgical treatment. Sociodemographics explained 2%, PROMs for physical and mental health explained 2%, illness perception explained 16%, and treatment characteristics explained 5%. For the outcome expectations of patients scheduled for surgical treatment, the most-definitive model explained 14% of the variance. Sociodemographics explained 2%, PROMs explained 2%, illness perception explained 8%, and treatment characteristics explained 2%.

**Fig. 3:**
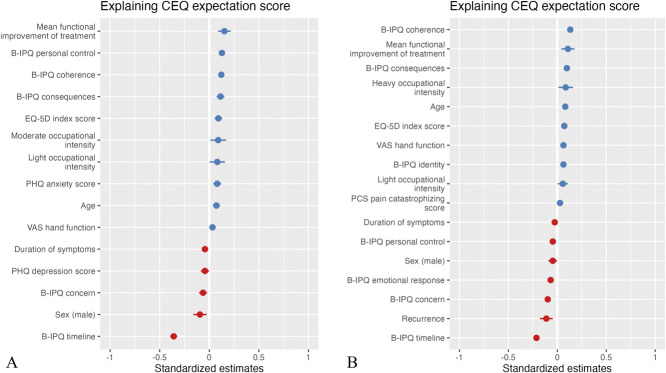
Standardized regression coefficients of the stratified hierarchical multilevel regression models explain outcome expectations for (**A**) nonsurgical treatment and (**B**) surgical treatment. Only significant variables are shown. EQ-5D = EuroQol-5 Dimensions; B-IPQ = Brief Illness Perception Questionnaire; PCS = Pain Catastrophizing Scale; PHQ = Patient Health Questionnaire. A color image accompanies the online version of this article.

When comparing the factors associated with outcome expectations between patients scheduled for nonsurgical treatment and those scheduled for surgical treatment, we found greater personal control was associated with more-positive expectations in nonsurgical patients (0.13; p < 0.001), whereas higher personal control was associated with more-negative expectations in surgical patients (-0.05; p < 0.001) (Fig. 3). Psychological distress was associated with expectations only in nonsurgical patients (depression: -0.04; p = 0.04; anxiety: 0.08; p < 0.001). Pain catastrophizing (0.03; p = 0.03), whether the patient has been treated for the same disease before (-0.11; p = 0.001), and a larger number of symptoms the patient views as being part of their Illness (0.06; p < 0.001) were associated with expectations only in surgical patients (Table 3).

## Discussion

Multiple studies have shown that more-positive outcome expectations are associated with better treatment outcomes [1, 5, 6, 8, 23, 27, 33], and there is an interest in positively modifying outcome expectations to improve treatment outcomes. However, little was known about factors independently associated with outcome expectations. Studying factors associated with outcome expectations may provide relevant information for clinicians and researchers aiming to improve outcome expectations. Improving expectations might, in turn, improve treatment outcomes. We found patients’ outcome expectations for a hand or wrist condition were higher when patients had more-positive perceptions of their illness. Furthermore, patients scheduled for surgical treatment had higher outcome expectations than patients scheduled for nonsurgical treatment, even after adjusting for differences in clinical profile and mindset between patents. Our findings can be used directly in daily clinic by improving expectations and illness perceptions, especially for nonsurgical patients, or in studies that develop interventions to improve expectations.

### Limitations

Our study has several limitations. First, because this was an observational study, no causal conclusions can be drawn. Although we theorized the variables in our model drive outcome expectations, the reverse could be just as true for several variables (outcome expectations may be causing illness perceptions), or instead, the relationship may be bidirectional. Experimental studies are necessary to test whether outcome expectations might be strengthened by influencing illness perceptions. Second, we found small differences between patients who responded to the survey (responders) and those who did not (nonresponders). Nonresponders were more often scheduled for nonsurgical treatment and had lower expectations. This is in line with other studies that showed nonsurgical patients are more likely to be lost to follow-up than surgical patients [8, 16, 36]. Furthermore, our study and others showed that patients scheduled for nonsurgical treatment have lower expectations [13, 26, 36], so we may assume the difference in expectations between responders and nonresponders is caused by the difference in treatment type we found in the nonresponder analysis. Still, we may have overestimated the expectations of patients undergoing nonsurgical treatment in our study. Third, our study examined pretreatment expectations, but several studies suggested outcome expectations may change during treatment and this change may influence treatment outcomes [11, 26]. Nevertheless, a robust association between pretreatment outcome expectations and treatment outcomes has been found in several medical areas [1, 5, 6, 27], indicating the importance of addressing pretreatment expectations. Future research could investigate whether the extent to which outcome expectations change during treatment depends on the type of treatment and how this change affects outcome.

### Association of Location, Surgeon, and Treatment Variables With Outcome Expectations

Nineteen percent of the variance in outcome expectations was attributable to differences between treatments rather than differences within treatments. Considering the surgeon and location level, we found the variance in outcome expectations was because of differences in surgeon or location, and almost none was because of differences between surgeons or locations. Theoretically, a surgeon adjusts his or her behavior to the patient, treatment, or other factors, such as workload. This might explain why we mainly saw within-surgeon differences.

### Patient and Treatment Factors Independently Associated With Outcome Expectations

Our study showed illness perception is an important factor strongly associated with outcome expectations. The more positively patients perceived their illnesses, the more positive their expectations were of the treatment outcome. Perceived chronicity of the disease and the perceived understanding of the disease displayed the strongest independent association. Given studies usually investigate variables associated with outcome expectations of a single treatment, previous researchers may have missed an important overarching factor driving expectations: the type of treatment a patient is about to undergo. In our study, approximately 18% of the total variance across patients was explained by the treatment invasiveness (nonsurgical, minor, or major surgical treatments) and the past effectiveness of the treatment. These results might indicate that patients believe treatment invasiveness is positively associated with better outcomes, resulting in higher pretreatment expectations by patients scheduled for surgical treatment. This finding is in line with those of other studies [16, 17, 36]. Our study indicates that expectation management should be tailored to the specific treatment (surgical or nonsurgical) and to the specific patient (including their perception of illness). For example, an intervention aimed to increase the understanding of a specific illness and accompanying treatment (such as offering an illness-specific or patient-specific e-learning module with psychoeducation to provide information and support so a patient will better understand their illness and treatment) might effectively correct false (negative) beliefs regarding treatment invasiveness in nonsurgical patients and thus improve their pretreatment expectations.

We found an association with the treatment effectiveness based on the mean improvement in function in historical patients, but not for the mean improvement in pain. Hypothetically, in their explanation of treatment effectiveness, clinicians might avoid strong statements about pain, because the amount of improvement in pain differs greatly between patients and between treatments. However, statements on hand function, including a statement such as “you will be able to return to work within 12 weeks,” might be safer because this outcome may be more predictable. Additionally, we did not find an association between the amount of pain at baseline, whereas for function, we found patients with better pretreatment function had higher expectations. This suggests pain might be less important for outcome expectations than pretreatment level of function is.

### Differences Between Patients Scheduled for Nonsurgical Treatment and Those Scheduled for Surgical Treatment

The degree of control patients feel they have over their illness was the only illness perception domain not associated with outcome expectations in our hierarchical regression model. However, our stratified analysis shows that the more personal control a nonsurgical patient experienced, the more positive the outcome expectations were, whereas the reverse was true for surgical patients. Because of this opposite effect, they may likely have cancelled each other out in the overall regression analysis. This opposite effect might guide intervention for improving outcome expectations. Patients with an internal locus of control perceive themselves as having a great deal of personal control over their outcomes, whereas patients with an external locus of control believe their outcomes result from external influences. Considering the locus of control, improving outcome expectations in nonsurgical patients should entail an increase in personal control (such as a greater understanding of illness and self-efficacy). In contrast, the outcome expectations of surgical patients might be improved by discussing important external influences (including physician experience and the likelihood of success with treatment).

### Conclusion

So far, there is some promising evidence for expectancy-focused interventions to improve treatment outcomes [32]. Expectation management appears to be an important element of delivering high-quality healthcare. Our findings suggest expectation management should be tailored to the specific treatment (such as surgical versus nonsurgical) and the specific patient (including their perception of their illness). It may be more beneficial to test and implement expectation management strategies such as physical therapy for nonsurgical treatments than for surgical treatments, given our findings indicate a greater need to do so. An additional advantage of such a strategy is that successful interventions may be able to prevent converting to surgical interventions, which is a goal of the stepped-care principles of standard care. Future studies might investigate the association between pretreatment expectations and outcomes by performing an experimental study, such as a randomized controlled trial, in which boosting expectations is compared with usual care (with no special attention to expectations) in nonsurgical and surgical groups.

## Group Authors

Members of the Hand-Wrist Study Group include: Richard Arjen Michiel Blomme, Dirk-Johannes Jacobus Cornelis van der Avoort, Alexander Kroeze, Jeronimus Maria Smit, Jan Debeij, Erik Taco Walbeehm, Gijs Marijn van Couwelaar, Johannes Pieter de Schipper, Johannes Frederikes Maria Temming, Jeroen Hein van Uchelen, Herman Luitzen de Boer, Kennard Harmsen, Oliver Theodor Zöphel, Thybout Matthias Moojen, Xander Smit, Gert-Jan Halbesma, Rob van Huis, Pierre Yves Alain Adriaan Pennehouat, Karin Schoneveld, Yara Eline van Kooij, Joris Jan Veltkamp, Alexandra Fink, Joris Sebastiaan Teunissen, Jaimy Emerentiana Koopman, Nina Louisa Loos, Jak Dekker, Ward Rogier Bijlsma, and Marloes Hendrina Paulina ter Stege
